# Metformin Use Correlates with Reduced Risk of Gallstones in Diabetic Patients: A 12-Year Follow-up Study

**DOI:** 10.3389/fphar.2017.00765

**Published:** 2017-10-24

**Authors:** Kuan-Fu Liao, Hsun-Yang Chuang, Shih-Wei Lai

**Affiliations:** ^1^College of Medicine, Tzu Chi University, Hualien, Taiwan; ^2^Department of Internal Medicine, Taichung Tzu Chi General Hospital, Taichung, Taiwan; ^3^College of Medicine, China Medical University, Taichung, Taiwan; ^4^Department of Family Medicine, China Medical University Hospital, Taichung, Taiwan

**Keywords:** diabetes mellitus, gallstones, metformin, Taiwan, National Health Insurance Program

## Abstract

**Objective:** Few studies are available on the association between gallstones and metformin use. The objective of the study was to determine whether metformin use is associated with gallstones.

**Methods:** A population-based retrospective cohort study was conducted using the database of the Taiwan National Health Insurance Program. Subjects of newly diagnosed diabetes mellitus were included from 2002 to 2013. The metformin-exposure group was defined as ≥29 cumulative defined daily dose (DDD) of metformin use. The un-exposure group was defined as <29 cumulative DDD of metformin use. The major endpoint was a new diagnosis of gallstones during the follow-up period. A multivariable Cox proportional hazards regression model was used to evaluate the hazard ratio (HR) and 95% confidence interval (CI) of gallstones associated with metformin use.

**Results:** After controlling for potential confounders, the adjusted HRs of gallstones were 1.11 (95%CI: 0.84–1.46) for subjects with metformin dosage of 29–180 cumulative DDD, and 0.57 (95%CI: 0.42–0.78) for subjects with metformin dosage >180 cumulative DDD, compared with the un-exposure group.

**Conclusion:** Long-term use of metformin is associated with reduced risk of gallstones.

## Introduction

Diabetes mellitus remains a public health problem in Taiwan due to its high incidence and prevalence, and significant mortality (Liao and Tsai, 2014; Jao et al., 2015; Yang et al., 2017). One epidemiological study using the database of Taiwan National Health Insurance Program has shown that the incidence and the prevalence of diabetes mellitus gradually increased in Taiwan from 2000 to 2008 (Jiang et al., 2012). Overall, the increase of the incidence was 14.6% for women and 29.6% for men from 2000 to 2008 (Jiang et al., 2012). The increase of the prevalence was 56% for women and 74% for men from 2000 to 2008 (Jiang et al., 2012). Diabetes mellitus was the fifth leading cause of death in Taiwan in 2015 (crude mortality rate 40.6/per 100000 persons) (Ministry of Health and Welfare, Taiwan, 2015). Diabetes mellitus has been associated with increased risk of some comorbidities including microvascular and macrovascular complications, and cancer (Bailes, 2002; Pandey et al., 2011; Shikata et al., 2013; Forouhi and Wareham, 2014). One meta-analysis has shown that diabetes mellitus is associated with increased risk of gallbladder disease including gallstones, cholecystectomy, and cholecystitis (relative risk 1.56, 95% CI: 1.26–1.93) (Aune and Vatten, 2016).

Metformin is the first-line medication to treat diabetes mellitus. To date, no large scale study explores the association between gallstones and metformin use. Therefore, a population-based retrospective cohort study was conducted using the database of the Taiwan National Health Insurance Program to explore the association between gallstones and metformin use.

## Materials and Methods

### Ethics Statement

The Institutional Review Board of Taichung Tzu Chi General Hospital in Taiwan approved the study protocol (REC104-30). Because the identification numbers and personal information of the individuals in the study were not included in the secondary files, the review board waived the need for written consent.

### Data Source, Study Design, and Subject Selection

Taiwan is an independent country with more than 23 million people (Li et al., 2015; Lin C.H. et al., 2015; Lin C.J. et al., 2015; Lin T.Y. et al., 2015; Lin Y.J. et al., 2015; Liu J.C. et al., 2015; Liu S.P. et al., 2015; Liu W.H. et al., 2015; Su, 2015; Tien et al., 2015; Wu C.Y. et al., 2015; Wu I.C. et al., 2015; Yao et al., 2015; Yin, 2015; Lai et al., 2017b; Liao et al., 2017b). A population-based retrospective cohort study was conducted using the database from the Taiwan National Health Insurance Program. The database is available to researchers (National Health Insurance Research Database, 2017).

Subjects of newly diagnosed diabetes mellitus (International Classification of Diseases, Ninth Revision, Clinical Modification, ICD-9 code 250) were included from 2002 to 2013. The date for subjects being diagnosed with diabetes mellitus was defined as the index date. Subjects who had a diagnosis of gallstones, or had at least a prescription for metformin before the index date were excluded from the study.

### Major Endpoint

It was a new diagnosis of gallstones during the follow-up period.

### Comorbid Conditions and Socioeconomic Status

Comorbid conditions and socioeconomic status were included as follows: atrial fibrillation, chronic kidney disease, coronary artery disease, heart failure, hypertension, lipid disorders, socioeconomic status, urbanization, and geographic region of residence. All comorbid conditions were diagnosed based on ICD-9 codes, which have been discussed in previous studies (Hung et al., 2011; Shen et al., 2016; Lai et al., 2017a; Liao et al., 2017a; Lin et al., 2017a,b).

### Assessment of Metformin Exposure

The definition of defined daily dose (DDD) was adapted from previous studies as follows (Lu et al., 2005; Yang et al., 2014; Lee et al., 2016). The DDD is regarded as a unit for estimating a prescribed amount of a medication. It is the assumed average daily maintenance dose of a medication prescribed for its major indication in adults. The cumulative DDD, which indicates the exposure duration of a medication, was estimated as the sum of dispensed DDD of metformin to compare its use with the risk of gallstones. The metformin-exposure group was defined as ≥29 cumulative DDD of metformin. The un-exposure group was defined as <29 cumulative DDD of metformin.

Other anti-diabetic medications were included as follows: dipeptidyl peptidase-4 inhibitor, insulin, sulfonylurea, and thiazolidinedione.

### Statistical Analysis

SPSS version 19 (SPSS Inc., Chicago, IL, United States) was used for all data analyses. Pearson’s chi-square test was used for categorical variables such as sex, other anti-diabetic medications use, comorbid conditions, socioeconomic status, urbanization, and geographic region of residence. Continuous variables were analyzed using one-way analysis of variance (ANOVA). A multivariable Cox proportional hazards regression model was used to evaluate the hazard ratio (HR) and 95% confidence interval (CI) of gallstones associated with metformin use after adjustment for subject characteristics (including age, sex, other anti-diabetic medications, comorbid conditions, socioeconomic status, urbanization, and geographic region of residence). Statistical significance was set at a two-sided *P* < 0.05.

## Results

### Characteristics of the Study Population

**Table 1** reveals the baseline characteristics between the metformin-exposure group and the un-exposure group. The study included 10182 subjects in the metformin-exposure group and 10182 subjects in the un-exposure group, with similar distributions of age and sex. The proportions of other anti-diabetic medications use, hypertension, and lipid disorders were statistically higher in the metformin-exposure group than the un-exposure group (Pearson’s chi-square test, *P* < 0.05).

**Table 1:** Baseline characteristics of the study population.

Variable	Exposure to metformin	Un-exposure	*P*-value
Subjects number	10182	10182	
Mean age, years (±standard deviation)	55 ± 21	55 ± 21	NA
**Sex**			NA
Male	5095 (50.0)	5095 (50.0)	
Female	5087 (50.0)	5087 (50.0)	
**Other anti-diabetic drugs**			
Dipeptidyl peptidase-4 inhibitor	2887 (28.4)	576 (5.7)	<0.001
Insulin	4252 (41.8)	2460 (24.2)	<0.001
Sulfonylurea	8300 (81.5)	3809 (37.4)	<0.001
Thiazolidinedione	2593 (25.5)	505 (5.0)	<0.001
**Comorbidities**			
Atrial fibrillation	139 (1.4)	179 (1.8)	0.024
Chronic kidney disease	43 (0.4)	230 (2.3)	<0.001
Coronary artery disease	903 (8.9)	977 (9.6)	0.073
Heart failure	329 (3.2)	363 (3.6)	0.189
Hypertension	3770 (37.0)	3375 (33.1)	<0.001
Lipid disorders	1482 (14.6)	1348 (13.2)	0.007
**Socioeconomic status**			0.581
Low socioeconomic status	4425 (43.5)	4386 (43.1)	
Moderate and high socioeconomic status	5757 (56.5)	5796 (56.9)	
**Urbanization**			0.128
Urban	2839 (27.9)	2937 (28.8)	
Un-urban	7343 (72.1)	7245 (71.2)	
**Geographic region of residence**			0.063
Northern/Central	6643 (65.2)	6769 (66.5)	
Southern/Eastern	3539 (34.8)	3413 (33.5)	


*The metformin-exposure group was defined as ≥29 cumulative defined daily dose (cumulative DDD) of metformin use. The un-exposure group was defined as<29 cumulative DDD of metformin use.*

### Association of Gallstones with Metformin Use

After adjustment for subject characteristics (including age, sex, other anti-diabetic medications, comorbid conditions, socioeconomic status, urbanization, and geographic region of residence), a multivariable Cox proportional hazards regression model revealed that the adjusted HRs of gallstones were 1.11 (95%CI: 0.84–1.46) for subjects with metformin dosage of 29–180 cumulative DDD, and 0.57 (95%CI: 0.42–0.78) for subjects with metformin dosage >180 cumulative DDD, compared with the un-exposure group (**Table 2**).

**Table 2:** Hazard ratio (HR) and 95% confidence interval (CI) of gallstones associated with metformin use in a multivariable Cox proportional hazard model.

Metformin use	Gallstones	
	
	HR(95%CI)	*P*-value
Un-exposure	(Reference)	
Metformin dosage of 29–180 cumulative DDD	1.11 (0.84–1.46)	0.469
Metformin dosage >180 cumulative DDD	0.57 (0.42–0.78)	<0.001


*HR, hazard ratio; CI, confidence interval; Adjusted for age, sex, dipeptidyl peptidase-4 inhibitor, insulin, sulfonylurea, thiazolidinedione, atrial fibrillation, chronic kidney disease, coronary artery disease, heart failure, hypertension, lipid disorders, socioeconomic status, urbanization, and geographic region of residence.*

## Discussion

We found that metformin use was associated with reduced risk of gallstones for subjects with metformin dosage >180 cumulative DDD (adjusted HR 0.57, **Table 2**). However, the HR was 1.11 for subjects with metformin dosage of 29–180 cumulative DDD, without statistic significance. These findings indicate that there is a duration-response relationship between the risk of gallstones and metformin use. That is, the protective effect on gallstones is greater for longer duration of metformin use.

The mechanisms underlying the protective effect of metformin use cannot be fully determined in our observational study. Moreover, De Santis et al.’s (1997) study has shown that the fasting glucose levels and 2-h glucose levels of oral glucose tolerance test were significantly higher in patients with gallstones than those without gallstones. As well known, hemoglobin A1c is an important measure of glycemic control. It has been a better indicator of long-term glycemic control. Al-Bayati and Kodayer’s (2012) study has shown that the prevalence of gallstones was higher among patients with high level of hemoglobin A1c. That is, high glycemic status is associated with a high prevalence of gallstones (De Santis et al., 1997; Al-Bayati and Kodayer, 2012). Therefore, we make a rational explanation that patients with long-term use of metformin could have a good glycemic control, which further reduce the risk of gallstones development. The present study cannot prove this issue because hemoglobin A1c was not recorded due to the inherent limitation of the database. However, it indicates a further research direction on the association between gallstones and hemoglobin A1c levels.

Some limitations should be discussed. Theoretically, we need to test the association between gallstones and medications for comorbidities. It is difficult to conduct such a cohort study which needs to include all concomitant medications for adjustments. The rational step is to test the relative risk of gallstones associated with one potential drug. According to the recommendation of American Diabetes Association (2017) metformin is the first-line medication for patients with type 2 diabetes mellitus. That is why we only included metformin for statistical analysis. However, it indicates a further research direction on the association between gallstones and other concomitant medications.

Some strength should be discussed. To the best of our knowledge, this is the first epidemiological study to explore the association between gallstones and metformin use. This is a unique finding supported by a large database.

## Conclusion

We conclude that long-term use of metformin is associated with reduced risk of gallstones. A further research is needed to clarify the association between gallstones and hemoglobin A1c levels.

## Author Contributions

K-FL contributed to the conception of the article, participated in the data interpretation, and revised the article. H-YC conducted the data analysis and revised the article. S-WL initiated the draft of the article and revised the article.

## Conflict of Interest Statement

The authors declare that the research was conducted in the absence of any commercial or financial relationships that could be construed as a potential conflict of interest.
